# Supplementary material for: Gait signature changes with walking speed are similar among able-bodied young adults despite persistent individual-specific differences
Source: bioRxiv. 2024 May 3:2024.05.01.591976. Preprint. [Version 1] doi: 10.1101/2024.05.01.591976 (PMC11092667; doi:10.1101/2024.05.01.591976)
Supplement: Supplement 1 [file NIHPP2024.05.01.591976v1-supplement-1.pdf]

bioRxiv preprint doi: https://doi.org/10.1101/2024.05.01.591976; this version posted May 3, 2024. The copyright holder for this preprint (which was not certified by peer review) is the author/funder, who has granted bioRxiv a license to display the preprint in perpetuity. It is made available under aCC-BY 4.0 International license.

6 Supplementary Figures

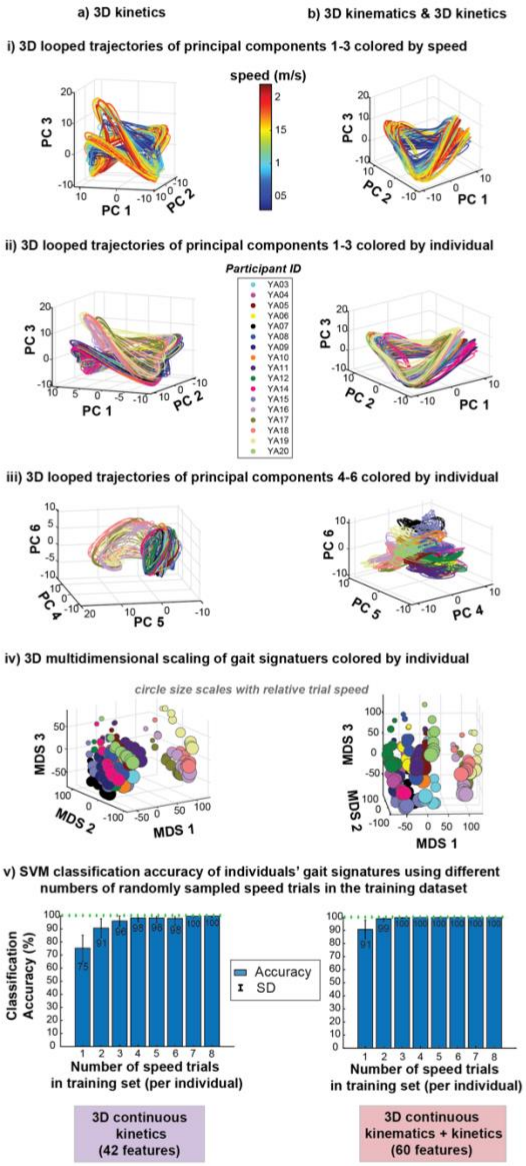

Supplementary Fig. S1: Visualization of kinetic-based gait signatures a) 3D kinetics and b) 3D kinematics & 3D kinetics. i) 3D looped trajectories of the first 3 principal components (PCs 1-3) of the gait signatures colored by speed shows that gait signatures at faster speeds (red) were more expansive than those at slower speeds (blue) ii) 3D looped representations of the first 3 PCs 1-3 of the gait signatures colored by individuals revealed that signatures are individual-specific across speeds. Kinetic signatures (a) appeared to form 2 groups of individuals with differing looped trajectories. iii) 3D looped representations of the second set of PCs 4-6 of the gait signatures colored by individual showcased individual-specific signatures. iv) 3D MDS visualizations of all signatures colored by individual further reveals a splitting of individuals into two groups. v) Individual classification accuracy was relatively high using a) 3D kinetics and b) 3D kinetics & kinematics across varied number of speed trials in the classification model training set.

bioRxiv preprint doi: https://doi.org/10.1101/2024.05.01.591976; this version posted May 3, 2024. The copyright holder for this preprint (which was not certified by peer review) is the author/funder, who has granted bioRxiv a license to display the preprint in perpetuity. It is made available under aCC-BY 4.0 International license.

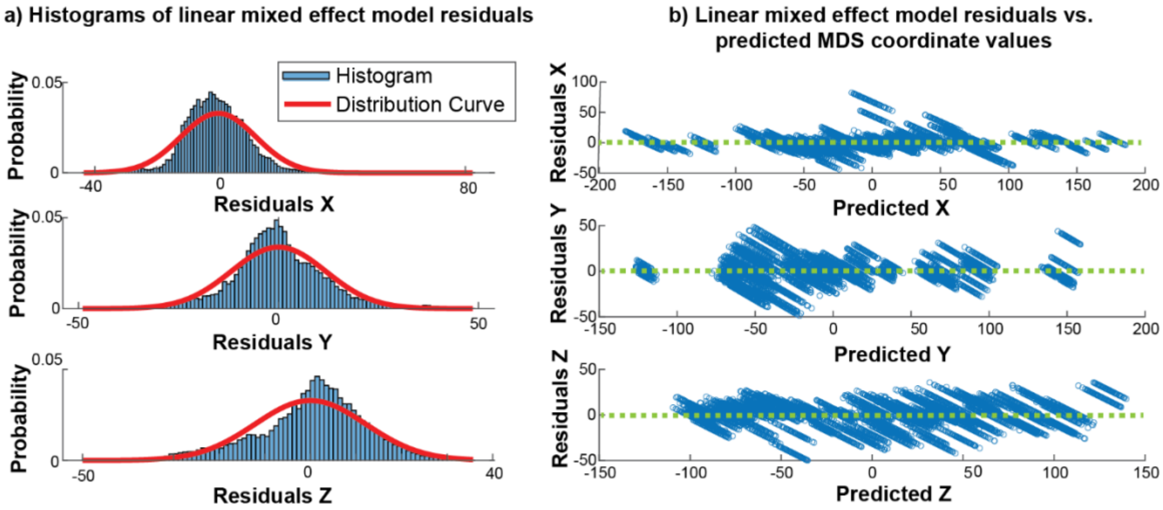

Supplementary Fig. S2: Evaluation of LME model fits. a) Histogram of residuals across 3D coordinate LME models are centered around zero. b) Residuals vs. predicted values reveal homoscedasticity (fluctuation around zero) regardless of prediction value.

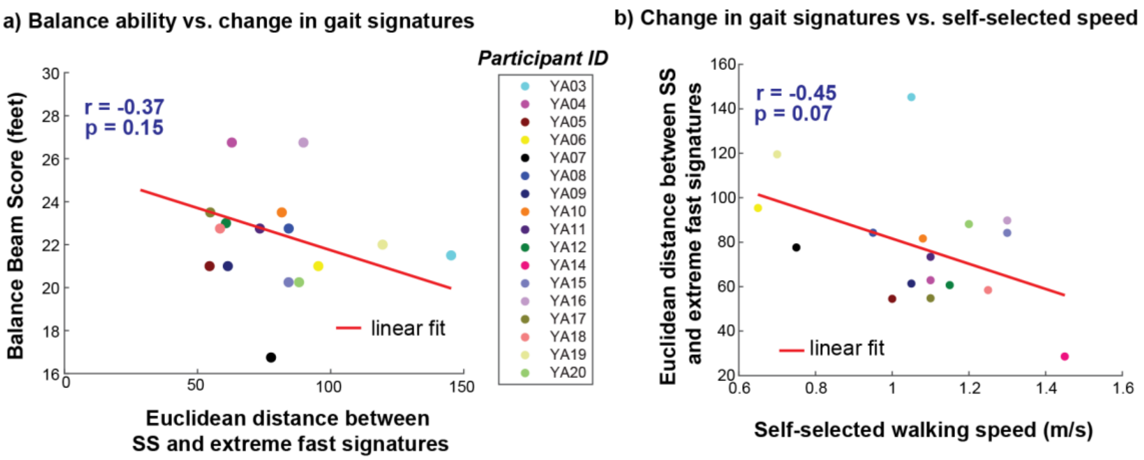

Supplementary Fig. S3: Correlation plots showing no significant linear relationships between Euclidean distance between SS and extreme fast (walk to run transition) speed signatures and a) narrowing balance beam score and b) self-selected walking speed.

bioRxiv preprint doi: https://doi.org/10.1101/2024.05.01.591976; this version posted May 3, 2024. The copyright holder for this preprint (which was not certified by peer review) is the author/funder, who has granted bioRxiv a license to display the preprint in perpetuity. It is made available under aCC-BY 4.0 International license.

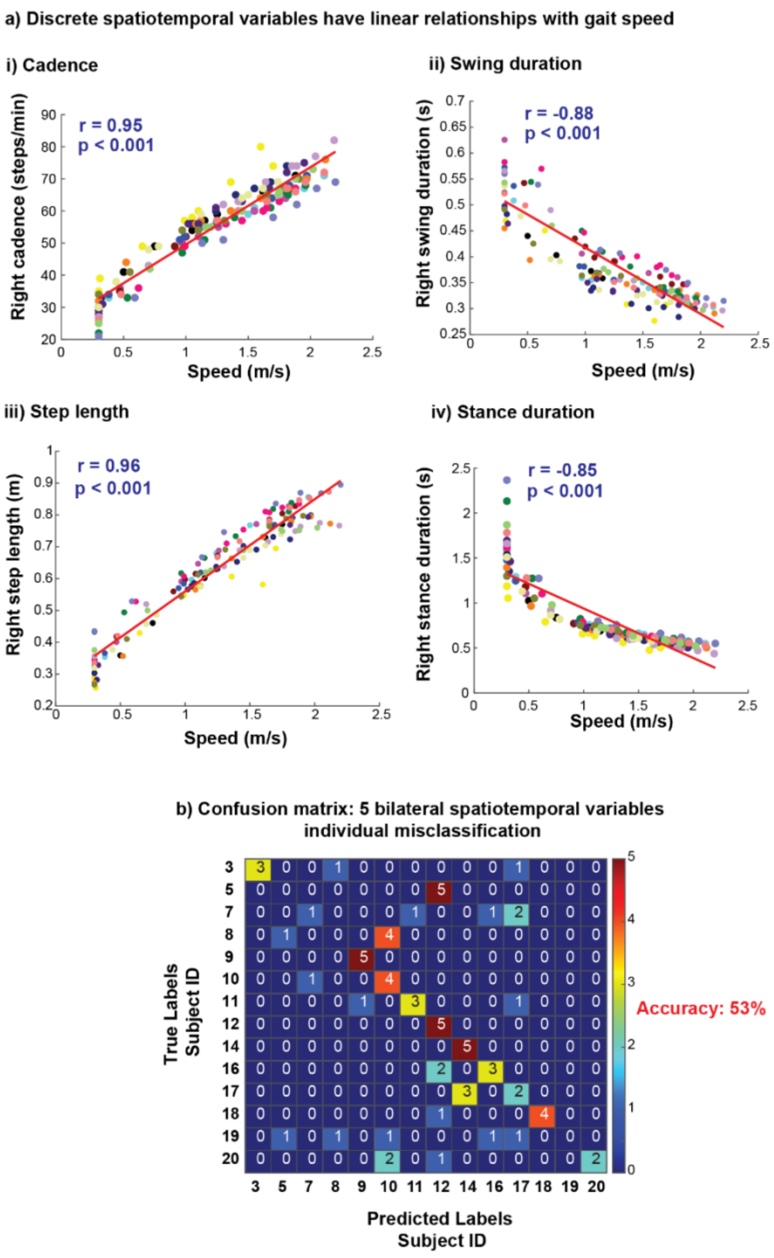

Supplementary Fig. S4: Discrete biomechanical variables show strong, linear relationships with speed. a) Discrete spatiotemporal variables i) cadence and iii) step length show strong positive linear relationships with increasing gait speed and variables ii) swing duration and iv) stance duration show strong negative linear relationships with increasing gait speed. b) Five bilateral spatiotemporal discrete variables (cadence, step length, swing duration, stance duration and step width) were unable to classify individuals with high accuracy (53%). A confusion matrix, derived from a single run of a linear support vector machine classification model, illustrates that multiple individuals were misclassified.

bioRxiv preprint doi: https://doi.org/10.1101/2024.05.01.591976; this version posted May 3, 2024. The copyright holder for this preprint (which was not certified by peer review) is the author/funder, who has granted bioRxiv a license to display the preprint in perpetuity. It is made available under aCC-BY 4.0 International license.

Supplementary Table. T1: 13 commonly used discrete biomechanical variables assessed bilaterally in gait analysis

Kinematics	Kinetics
Step length	Peak anterior ground reaction force
Peak trailing limb angle	Push off integral
Peak hip hike	Peak ankle moment
Double support duration	Peak ankle power
Stance duration	
Swing duration	
Ankle angle at heel strike	
Ankle angle at toe off	
Knee angle at midstance	
